# Lessons learned about psychosocial responses to disaster and mass trauma: an international perspective

**DOI:** 10.3402/ejpt.v4i0.22897

**Published:** 2013-12-20

**Authors:** Lennart Reifels, Luca Pietrantoni, Gabriele Prati, Yoshiharu Kim, Dean G. Kilpatrick, Grete Dyb, James Halpern, Miranda Olff, Chris R. Brewin, Meaghan O'Donnell

**Affiliations:** 1 Centre for Mental Health, Melbourne School of Population and Global Health, The University of Melbourne, Melbourne, Australia; 2 Department of Psychology, University of Bologna, Bologna, Italy; 3 School of Political Science, University of Bologna, Bologna, Italy; 4 National Center of Neurology and Psychiatry, Department of Adult Mental Health, Tokyo, Japan; 5 Department of Psychiatry and Behavioral Sciences, Medical University of South Carolina, Charleston, SC, USA; 6 Norwegian Centre for Violence and Traumatic Stress Studies, University of Oslo, Oslo, Norway; 7 Institute for Disaster Mental Health, State University of New York, New Paltz, NY, USA; 8 Academic Medical Center, University of Amsterdam, Amsterdam, The Netherlands; 9 Arq Psychotrauma Expert Center, Diemen, The Netherlands; 10 Clinical, Educational & Health Psychology, University College London, London, United Kingdom; 11 Australian Centre for Posttraumatic Mental Health, Department of Psychiatry, The University of Melbourne, Melbourne, Australia

**Keywords:** Disasters, mental health, psychosocial, trauma, disaster planning

## Abstract

At the 13th meeting of the European Society for Traumatic Stress Studies in 2013, a symposium was held that brought together international researchers and clinicians who were involved in psychosocial responses to disaster. A total of six disasters that occurred in five countries were presented and discussed. Lessons learned from these disasters included the need to: (1) tailor the psychosocial response to the specific disaster, (2) provide multi-dimensional psychosocial care, (3) target at-risk population groups, (4) proactively address barriers in access to care, (5) recognise the social dimensions and sources of resilience, (6) extend the roles for mental health professionals, (7) efficiently coordinate and integrate disaster response services, and (8) integrate research and evaluation into disaster response planning.

The provision of psychosocial support to disaster-affected populations has been recognised as a key strategy in mitigating the adverse mental health effects of natural and man-made disasters (Bonanno, Brewin, Kaniasty, & LaGreca, 2010; Norris, Friedman, & Watson, 2002; North & Pfefferbaum, 2013; Ritchie, Watson, & Friedman, 2006). Guidelines for psychosocial disaster responses emphasise the need for multilevel support strategies to be provided in alignment with the needs and circumstances of affected populations (Bisson et al., 2010; Inter-Agency Standing Committee, 2007). These strategies range from provision of practical help; community-based interventions; and low-intensity supports (such as Psychological First Aid) to more specialised and intensive mental health treatments (such as trauma-focussed cognitive behavioural therapy, or TF-CBT; eye movement desensitisation and reprocessing therapy, or EMDR; or pharmacotherapy) for a minority of individuals who go on to develop severe mental health issues.

Despite the availability of disaster response guidelines and a growing evidence base for the efficacy of high-intensity interventions, each disaster event produces unique impacts and challenges that require the tailoring of psychosocial responses to the existing community, service systems, and disaster context. In view of these challenges, little is known about implementation frameworks that facilitate the translation of available support strategies for disaster-affected populations. Insights from international disaster responses therefore can provide valuable lessons to inform and improve future psychosocial disaster response planning (EFPA, 2009).

At the 13th meeting of the European Society for Traumatic Stress Studies in 2013, an ISTSS/ESTSS symposium was held that was chaired by Professors Miranda Olff and Chris Brewin. This symposium, “Improving Psychosocial Responses to Disaster and Mass Trauma: An International Perspective,” featured many researchers who had hands-on experience of dealing with a disaster. Within these presentations, a number of different disasters were explored, from natural disasters, like the 2011 Japan earthquake/tsunami, the 2012 Northern Italian earthquakes, and the 2004 Florida hurricanes, to man-made disasters, including the 2005 London bombings and 2011 Norwegian terrorist mass shooting. In addition to this symposium, insights were gleaned from the response to the 2012 US Sandy Hook, Newtown, school shooting. In this paper, we briefly describe these disasters, the psychosocial responses delivered, key findings from any research undertaken, and lessons learned from the disaster. This is followed by a discussion on a number of key themes that emerged from the disaster responses described within the presentations.

## The 2012 Northern Italian earthquakes

Dr Pietrantoni presented an outline of the psychosocial response to the 2012 Northern Italian earthquakes and findings of survey research conducted in the disaster aftermath.

In May 2012, two major earthquakes struck the Italian region of Emilia Romagna that affected 14,350 people. These earthquakes caused 26 deaths, damaged 33,600 buildings, and resulted in widespread evacuation of affected community members. The psychosocial response to the disaster involved the development of self-help materials (e.g., leaflets, website), conduct of needs assessments, and the provision of practical support by local health authorities, non-government organisations (NGOs) (e.g., Red Cross, Save the Children), and private organisations to 13,000 people living in 52 temporary camps and emergency shelters. In addition, the local health authority of Modena provided targeted group counselling sessions to health care workers of the affected area aimed at mitigating acute distress and helping them in the decision-making process on relocation and organisational change. Individual psychotherapy (EMDR) was provided to symptomatic individuals in the weeks after the earthquake as an early intervention. Alongside these formal support measures, a significant community response involved the mobilisation of 2,000 volunteers, organisation of fundraising events, campaigns to “adopt schools,” and other initiatives in support of affected communities. Volunteers are usually mobilised from the community, and although they are classified as a resource for emergency response, they are also often affected community members who experience adverse effects themselves (Thormar et al., 2010).

### Research

An online survey (*N*=1,839) conducted by Dr Pietrantoni and colleagues immediately after the first of the two major earthquakes examined human reactions and behaviour during the shock (Prati, Saccinto, Pietrantoni, & Pérez-Testor, 2013). Study findings highlighted that anxiety and stress symptoms were commonly experienced among disaster victims. Moreover, the most frequent behavioural responses during the earthquake were those identified as unsafe, such as moving to another room of the house or attempting to leave the building.

### Lessons

Dr Pietrantoni identified three key lessons learned from the 2012 Northern Italy Earthquakes. First, earthquake preparedness programs were needed: most individuals were at risk for injuries and fatalities due to inappropriate or unsafe behavioural responses during the shocks, such as moving to another room (Prati et al., 2013). Second, there was some indication of the usefulness of psychosocial interventions to increase personal and organisational resilience (Florini, Guglielmi, Brunetti, Camellini, & Vignoli, 2012). However, it was noted that most of the psychological interventions were oriented to individuals, and less attention was devoted to the role of contextual and social dimensions. Future responses in these contexts could therefore be improved by focussing more strongly on resilience factors at family, organisation, and community levels. For example, given the protective role of social integration and social support, interventions should mobilise social support networks (e.g., family, friends, and community members) and promote collective action, participation, and empowerment (Prati & Pietrantoni, 2010). Third, there was recognition that psychosocial interventions were more effective when they focussed on the promotion of networking between different institutions and NGOs. For example, the organisation “Save the Children” successfully adopted this approach (De Bernart & Rivello, 2012; Piccinini, 2012).

## The 2011 Great East Japan earthquake, tsunami, and nuclear disaster

Dr Kim addressed the psychosocial response to the 2011 Great East Japan earthquake, tsunami, and nuclear disaster.

In March 2011, a major earthquake with an epicentre off the Japanese coast triggered a gigantic tsunami that affected large regions of North Eastern Japan and caused a nuclear accident at the Fukushima Daiichi power plant. The combined impact of the earthquake and tsunami resulted in 15,828 deaths, 6,145 injured and 4,823 missing people, and damage to more than 1 million buildings. The nuclear accident required the evacuation of whole regions due to radiation concerns. In response to this disaster, the National Center of Neurology and Psychiatry established a central website that made relevant disaster response information (including the Japanese guidelines on post-disaster mental health care, related manuals, and leaflets) available to mental health professionals and the wider public (Kim, 2011; Kim & Akiyama, 2011). The multi-dimensional care model underpinning the psychosocial response involved the provision of psychological first aid, psychoeducation, counselling and specialised treatments (such as pharmacotherapy and prolonged exposure therapy). Almost 60 multi-professional mental health care teams were deployed to disaster-affected prefectures by the Japanese Ministry of Health, Labor and Welfare in order to provide mental health care (Suzuki & Kim, 2012). An important challenge to overcome arose from the need to evacuate many psychiatric hospitals resulting in more than 1,000 inpatients requiring transportation to neighbouring facilities within 10 days of the disaster. An acute shortage of psychiatric drugs and logistic supply challenges (e.g., destroyed road networks and infrastructure) had to be addressed to ensure treatment and service continuity in afflicted areas. The integration of international support offers within local health systems and communities constituted another challenge. In many situations, well-intended but inappropriate international interventions had the unintended consequence of causing confusion and discouragement among local caregivers.

### Lessons

Dr Kim highlighted three key lessons from the response. The first concerned the remarkable resilience of the Japanese people in the face of this great adversity. It was recognised, however, that there was a need to increase community and individual autonomy for mastering mental health difficulties, and to provide support for mental as well as secondary social difficulty following disaster. The second lesson related to the important dual disaster response task and challenge of ensuring the continuity of existing mental health services for people with pre-existing mental health problems (such as schizophrenia) whilst striving to establish adequate supports for survivors who are at risk of developing newly emerging mental health issues post-disaster. The third lesson highlighted the importance of coherence and a shared understanding among the great number and diversity of organisations involved in the mental health response. In this context, the disaster response headquarters assumed an important tripartite role involving administrative, academic and information functions. Moreover, available national guidelines, a central information portal, regular provider briefings, and cross-professional work in mental health care teams provided key mechanisms to aid response coordination. Future disaster response planning for disasters of this magnitude may further need to incorporate effective mechanisms to capitalise on international disaster expertise whilst ensuring greater adherence to and better integration with existing national frameworks and guidelines for disaster response.

## The 2004 Florida hurricanes

Dr Kilpatrick presented findings from an epidemiological study conducted in the aftermath of the 2004 Florida Hurricanes and lessons in regard to a new extended role for mental health professionals in disaster responses.

Over the course of only 7 weeks between August and September 2004, four hurricanes (three of which were classified as major at landfall) tore through 38 counties in the state of Florida, USA. These hurricanes resulted in 124 deaths and tremendous damage to property and infrastructure across many counties (Acierno et al., 2007). The disaster response involved input from local, state, and national government agencies and NGOs (such as the American Red Cross), which provided a variety of services to meet basic material needs for food, water, shelter, and safety as well as short-term crisis-oriented mental health services.

### Research

As part of the Florida Hurricane Study, Kilpatrick and colleagues (2007) examined disaster mental health sequelae and gene–environment interactions in a household probability sample of 589 adult Florida residents. Study participants were interviewed at 6–9 months about levels of hurricane exposure, social support, post-disaster posttraumatic stress disorder (PTSD), and major depression, and provided saliva samples for genetic analysis. Outcome measures included DSM-IV diagnoses of post-disaster PTSD, GAD and MD, alcohol and tobacco use, and genotype variations in the serotonin transporter gene SLC6A4. Study findings showed that 10.9% of participants met criteria for at least one of the three disorders (PTSD, GAD, or MD), with significantly elevated levels of alcohol and tobacco use evident for participants meeting diagnostic criteria for these conditions versus those that did not. Genotype expression in itself did not have a significant main effect on the risk of mental disorders. However, a significant three-way interaction was found in which genotype expression moderated the risk of PTSD and MD under the high environmental stressor conditions of high hurricane exposure and low social support.

### Lessons

The Florida Hurricane Study demonstrated both the feasibility and benefits of appending genetic components to epidemiological disaster research. Research focussing on gene–environment interactions (not just main effects) can help us to better understand why exposure to environmental stressors has different effects in different people. Another lesson from the Hurricane response highlighted the extended scope for mental health professional involvement in disaster responses beyond the traditional role in providing crisis counselling, psychotherapy, or pharmacotherapy to disaster victims. Specifically, mental health professionals can advocate the use of research-based knowledge about disaster mental health problems and establish risk and protective factors to identify those most in need of assistance. They can also provide advice on the likely need for broad based basic and psychosocial services and, to a lesser extent, intensive mental health services to health professionals, disaster relief agencies, and public policy officials. The particular expertise of mental health professionals therefore lends itself as a resource to improving community disaster resilience, preparedness, and response planning and the delivery of post-disaster services.

## The 2005 London bombings

Dr Brewin presented an overview of the psychosocial response to the London bombings and findings from associated service research.

In July 2005, a series of suicide bombings occurred in central London that resulted in 52 deaths and 775 casualties, and which constituted the largest mass casualty event in the UK since World War II. The psychosocial response to the bombings included three phases: (1) the initial response of emergency and health services (focussed on immediate safety and survival); (2) the establishment of a humanitarian assistance centre (where advice, practical and emotional support, and counselling was provided to survivors on demand); and (3) the implementation of a trauma response program for a minority of survivors with persistent mental health problems. The latter phase, which was coordinated through a multi-agency steering group convened by the London Development Centre for Mental Health, involved the implementation of a screen and treat model, in which a centralised screening/outreach team provided advice to the public, identified and screened affected persons (using validated screening tools), and directed survivors to appropriate treatment as required. Additional clinical psychologists based at three specialist trauma centres provided nationally recommended treatment (TF-CBT or EMDR) involving local treatment protocols but standardised procedures and outcome measurement across centres (Brewin et al., 2008).

### Research

Service research conducted by Brewin et al. (2010) examined program usage, diagnoses, and outcomes in regard to the trauma response program. The majority of 910 program referrals were received during the first 7 months. Of 596 participants screened, 56% screened positive at some stage and received clinical assessment. Primary diagnoses included PTSD (69%), travel phobia (7%), and adjustment disorder (6%). The percentage of diagnosed participants referred to treatment increased steadily over the 2-year program duration. Clinically significant changes in post-traumatic and depression symptoms were noted for 66 and 56% of 104 treatment completers, respectively. Treatment gains were comparable to or exceeding those found in randomised controlled trials and were well maintained at 1 year (Brewin et al., 2010).

### Lessons

Three key lessons from the response to the London bombings were highlighted. First, the trauma response program delivered effective, evidence-based treatment and was found to be acceptable and appropriate by users. Second, notable barriers to program access included inflexible existing referral pathways, limited program familiarity of family doctors, low program usage among individuals approached by third parties, and challenges to survivor identification (such as institutional barriers to disclosing who had been affected). Third, in view of such barriers, disaster responses must not rely on normal referral pathways but can benefit from outreach and screening to improve access to care for survivors with longer-term needs who are otherwise likely to be overlooked. Moreover, some central organisation is highly desirable for identification of those involved in an emergency (e.g., a register: Close et al., 2013), for resolution of institutional conflicts and barriers, and for coordination of access to treatment. Structures and principles surrounding data protection and finance need to be in place before major incidents occur, not after.

## The 2011 mass shooting at Utøya Island, Norway

Dr Dyb presented an outline of the psychosocial response to the mass shooting at the Norwegian island of Utøya and findings from a related service research study.

On 22 July 2011, a mass shooting carried out by a single perpetrator at a Norwegian Labour Party youth summer camp on the island of Utøya resulted in 69 young people being killed, 56 hospitalised, and 500 survivors from all over the country directly affected. Beyond those directly affected, the mass shooting and preceding terrorist bombing in the city of Oslo also had a significant immediate impact on the broader Norwegian population (Dyb et al., 2013; Thoresen, Aakvaag, Wentzel-Larsen, Dyb, & Hjemdal, 2012;). The psychosocial response to the disaster, designed by the Norwegian Centre of Violence and Traumatic Stress Studies, involved the implementation of a proactive outreach and screening approach that integrated with existing mental health services across Norwegian municipalities. This approach involved the provision of telephone calls from local crisis teams to all affected youth and families. Designated crisis team contacts were responsible for monitoring the needs of survivors and families and providing practical help and support for at least one year, guided by the five essential elements of safety, calming, self and community efficacy, connectedness, and hope (Hobfoll et al., 2007). To identify people with emerging mental health needs, brief clinical screens were administered at 5–6 weeks, 3 months and 6 months. On the basis of identified mental health needs, contact was made with and treatment provided by existing primary care and specialist mental health services.

### Research

The first of three waves of the Utøya study, conducted by Dyb and colleagues, examined the implementation of response activities across municipalities, the relationship of service use to consumer need and risk factors, and consumer service perceptions. Of the 325 survivors interviewed at 4–5 months, the vast majority had experienced high levels of exposure to life threat, had been contacted proactively (with no significant differences evident in terms of age, gender, loss, exposure, PTSD, depression and anxiety variables), had a designated contact person, and had used specialised services of a psychologist or psychiatrist (Dyb, Jensen, Glad, Nygaard, & Thoresen, in press). Overall, only a small minority of interviewees reported unmet psychosocial needs at 4–5 months.

### Lessons

Three key lessons from the disaster response were highlighted by the presentation. First, early and proactive outreach following disaster should provide general support and resources to ease the transition back to normality, rather than providing unselected interventions to all survivors. Such an outreach approach should focus on practical help and pragmatic support, guided by established elements of early mass trauma intervention. Second, to ensure the continuity of support for survivors and families, designated contact persons can play important roles in monitoring the needs of survivors and facilitating access to relevant primary care and mental health services. The need for information, support and targeted interventions may fluctuate over time as trauma victims often experience secondary stressors, such as witnessing in criminal law trials, medical rehabilitation due to injuries, involvements in legal claims, extended media coverage of the event, and economic hardships that may influence fluctuations in distress over time. Hence, to capture these adverse changes in distress over time, outreach strategies should extend to at least the first year after trauma. Third, repeat administration of a brief screening instrument to disaster victims can facilitate identification of people with clinical needs and targeting of interventions, thus helping to ensure that all survivors who develop a need for services are identified and offered relevant attention.

## The 2012 Sandy Hook school shooting

Dr Halpern provided a first-hand account of his involvement as an American Red Cross worker and disaster mental health expert in the psychosocial response to the Sandy Hook school shooting in the USA.

On 14 December 2012, a 20-year-old man killed his mother, drove to the Sandy Hook elementary school in Newtown, Connecticut, killed 20 children (aged 5–7 years) and six adult educators, and then took his own life. The severe and sudden nature of the mass shooting left many affected community members in a state of shock, whilst creating a heightened sense of danger among the broader public. In response to the disaster, the American Red Cross provided practical assistance, psychoeducation, crisis and grief counselling to affected family members, first responders, and the wider community. At a basic level, crisis counsellors worked closely with State Troopers to help families to be and feel safe. Crisis counsellors played important roles in the context of death notifications (alongside state troopers and clergy members), in promoting access to existing social supports, supporting personal coping styles, and providing support when survivors met with officials. Psychoeducation was given to parents to assist them in how to support surviving children. Grief counselling needed to affirm the validity of different grieving styles within families and communities. Counsellors further needed to be culturally competent in working with different groups of first responders and faith-based communities.

### Lessons

Three important lessons from the disaster response were highlighted. First, immediate intervention or crisis counselling can be especially useful for those with risk factors which include (but are not limited to) experiencing death of a loved one or personal injury, or who are vulnerable because of their age. Early intervention could include: psychological first aid, advocacy, crisis counselling, referrals and public health messaging. Second, early intervention can help to promote a positive recovery environment by working with the “Maslow hierarchy of needs” from the bottom up, promoting safety, calm, self-efficacy, social support, and hope. Promoting a positive recovery environment may also involve protecting survivors from punitive or blaming others, or an intrusive press. Third, in the case of school shootings, counsellors can: work effectively with law enforcement after death notification to provide support and a perception of safety; provide calm, compassion, and cognitive support when survivors meet with officials; remind the caregivers of grieving children of the importance of reassurance, safety, routine, and honesty; and provide grief counselling, encouraging family members to tolerate each other's different grieving process as there is no right way to grieve or process a loss.

## Discussion

While symposium presentations differed significantly in terms of content and scope, and the level of intervention and research reported, and were at times based on anecdotal evidence from individual disaster experts, several key themes emerged from the presentations that may be of relevance to informing and improving future psychosocial disaster response planning. All of the disasters afflicted developed countries and resulted in a significant loss of life, a key risk factor for adverse mental health outcomes (Norris et al., 2002). However, the level of previous disaster exposure and preparedness varied considerably between affected countries and communities.

### Tailoring psychosocial disaster responses to the specific disaster

While the disasters shared many characteristics as potentially traumatic events, distinct variations in the type, scope, and population impact of these events and the structure of existing health service systems highlighted the need to tailor psychosocial responses. For example, the Norwegian mass shooting on the island of Utøya represented a “centrifugal disaster” which struck at a location where people had gathered temporarily, who then dispersed widely after the event (Lindy & Grace, 1986). As a consequence, the Norwegian response required national service coverage and integration with relevant local services (e.g., crisis teams, primary and specialist mental health care providers). By contrast, “centripetal disasters,” such as the Sandy Hook school shooting, which affect population groups in areas where they live, often require more localised responses, notwithstanding the need to recognise broader population impacts. Disasters of the scale of the Great Eastern Japan disaster with significant population, health care system and societal impacts may require the mobilisation of local, national and potentially international resources.

Another dimension to consider when tailoring the psychosocial response to the disaster is the type of the disaster—which is a significant predictor of post- and peri-traumatic outcomes (Grimm, Hulse, Preiss, & Schmidt, 2012). There is much evidence to suggest that in developed countries human-caused disasters, especially those that are intentional rather than accidental (including acts of mass-violence), are associated with more adverse mental health impacts than natural and technological disasters (Norris & Elrod, 2006). On the other hand, some natural disasters that destroy large parts of the infrastructure and lead to the break-up and dispersion of communities are also accompanied by very substantial mental health impacts (Galea et al., 2007). There are a variety of risk and resilience factors that need to be considered when planning the psychosocial response to the disaster (Bonanno et al., 2010).

### Provision of multidimensional care

Each of the disaster responses described above endorsed a multidimensional approach to psychosocial care ranging from the provision of broad-based low intensity support to specialised high-intensity interventions. This approach recognises the different needs for different groups of survivors across time. For example, in Japan this approach included the provision of early low-intensity initiatives, such as social support, psychological first aid, assessment, and psychoeducation, as well as high-intensity treatment to ensure those with serious mental illness could access appropriate medication. The American Red Cross response to the Sandy Hook school shooting provided practical assistance, psychoeducation, crisis and grief counselling to family members, first responders, and the wider community as well as referrals for those in need of long-term care.

### Targeting at-risk population groups

The identification and targeting of support at particular at-risk groups constituted a key task for disaster responses. All responses sought to target direct disaster survivors and other at-risk groups. For example, the Italian response provided practical support to displaced people living in camps and emergency shelters, and the American Red Cross targeted bereaved families directly affected by the school shooting. Survivor identification created a particular challenge in disaster contexts involving open public spaces (such as in the case of the London bombings). It is essential in these disaster contexts that a central organisation takes responsibility for the identification of those involved in the disaster (e.g., a survivor register). Such a register may provide a key mechanism for the identification of high-risk groups and effective targeting of services.

### Proactively addressing barriers in access to care

Barriers in access to care were commonly identified across presentations. These barriers included aspects associated with the disaster response such as the identification of survivors, limitations of existing referral pathways, destroyed infrastructure, and other difficulties in accessing appropriate care. Since barriers in the access to and utilisation of mental health care tend to be amplified following disaster (Elhai & Ford, 2009; Rodriguez & Kohn, 2008), psychosocial disaster responses need to proactively address these barriers in an effort to reach disaster victims (Gibson et al., 2006; Watson, Brymer, & Bonanno, 2011). Disaster responders adopted several strategies in order to address these limitations. The Japanese response involved deployment of mental health care teams to disaster-affected areas and the evacuation of psychiatric inpatients to neighbouring facilities. The responses to the London bombings and Norwegian mass shooting introduced a proactive outreach component to identify and engage disaster survivors. In addition, screening provided a mechanism to identify survivors in need of treatment and to target interventions. Public health messaging provided information on disaster mental health care and available services. Future attempts to improve service accessibility may need to take into account other factors (such as gender, age, disability, social–economic status, language, or culture) that can impact on access to disaster care (Dückers, 2013).

### Recognising the social dimensions and sources of resilience

The Italian and Japanese disaster responses highlighted that efforts to promote a positive recovery environment required literacy in the social and contextual dimensions of resilience and recovery (Silove & Steele, 2006). More specifically, the Italian earthquake response underlined the important place of community-level and self-help initiatives that needed to be recognised and fostered within disaster response planning alongside more formal psychosocial support strategies (Ajdukovic, 2004). Social support and bonding are important also to reduce negative psychobiological outcomes after trauma (Olff, 2012). One of the key tasks for disaster response planners therefore consists in recognising both the value of existing and emerging support networks of those affected by disaster (as well as their limitations) within a broader framework of psychosocial disaster care (McFarlane & Williams, 2012). In this context, community-and family-based supports and targeted capacity building initiatives deserve particular consideration (Hawe, 2009; Inter-Agency Standing Committee, 2007).

### Extended roles for mental health professionals

Lessons from the Florida hurricanes and Sandy Hook school shooting outlined the extended scope for mental health professional involvement in disaster responses. Such involvement generally required addressing the hierarchy of survivor needs from basic (e.g., safety) to higher (e.g., mental health) needs. For example, promoting a positive recovery environment in the context of the Sandy Hook shooting required protecting survivors from an intrusive press. Mental health professionals can work effectively alongside law enforcement in the context of death notifications. Other important roles in these contexts include survivor advocacy, fostering community disaster resilience, preparedness planning, and the design and implementation of public health messaging.

### Efficient coordination and integration of disaster response services

The efficient coordination and consistency of disaster response services was a key theme of many presentations. For example, existing national disaster mental health guidelines in Japan provided useful overarching frameworks to inform service provision and capacity building among varied professional groups and organisations (Kim, 2011). Central website portals provided access to disaster response guidelines, information for mental health professionals on evidence-supported interventions, and information for the wider public in terms of psychoeducation, self-help strategies, and services available. Accurate disaster response information and on-going provider briefings can facilitate public trust, response consistency, provider adherence to best practice, and assist in countering common misinformation following disaster.

Large-scale psychosocial disaster responses require coordinated efforts to address multiple competing demands in chaotic circumstances. These demands include the need to ensure the continuity of existing health services, establish enhanced psychosocial services for the disaster-affected population, coordinate response agencies, integrate international resources, monitor population disaster impacts, and outcomes of response services. To efficiently address these demands following disaster, it is vital that national frameworks for psychosocial disaster response exist that integrate firmly with prevailing health emergency and disaster response arrangements. Such frameworks need to clearly specify roles and responsibilities of agencies involved in emergencies and the allocation of financial and human resources. Designated lead agencies with clear lines of accountability and multi-agency steering groups can facilitate the integration of psychosocial response and recovery activities. Adequate disaster preparedness further requires access to a pool of qualified and trained providers who can provide evidence-supported interventions of varying intensity in the event of a disaster. Notwithstanding the need for consistency, disaster responses can provide opportunities for trialling innovative service models and act as catalysts for health system reform (WHO, 2013). The integration of psychosocial disaster response services with existing health services can increase their sustainability and facilitate a return to “normality” over time.

### Integrate research into disaster response planning

While we can learn much from psychosocial disaster responses, research is still required in moving many response components from evidence-informed to evidence-based. In particular, the efficacy of low- and medium-intensity interventions, such as Psychological First Aid, Skills for Psychological Recovery and crisis counselling, remains unknown (Forbes et al., 2010; North & Pfefferbaum, 2013; Shultz & Forbes, 2013). Given the difficulty of conducting research in the immediate disaster environment, there may be benefits in examining the efficacy of low- to medium-intensity interventions in non-disaster trauma settings. An alternative approach could involve establishing research protocols proactively in disaster-prone areas to facilitate rapid activation in the event of disaster and integration with future disaster responses.

Population-based needs assessments and health surveillance of high-risk groups remain of immense practical importance to estimating psychosocial service needs and targeting interventions following disaster. In addition, facility- and program-based research is required to monitor the implementation of disaster response services, including consumer access, uptake, and outcomes. Decades of disaster mental health research suggest that future epidemiological disaster studies need to incorporate novel aspects and theoretical perspectives in order to continue to be of scientific merit (Norris & Elrod, 2006), notwithstanding their undiminished practical relevance to informing disaster services.

It is important that we draw lessons from earlier catastrophes and integrate them in the service delivery to affected populations. Research in this field has largely neglected the opportunity to promote or, preferably, to speed up the adoption of lessons individual care providers and disaster planners can only learn after having experienced the work in chaotic contexts numerous times. Future research in this area could benefit from the explicit adoption of implementation science perspectives (Brownson, Colditz, & Proctor, 2012). Although there are still gaps between norms and practice (Te Brake & Dückers, 2013), the evidence-base for interventions is strengthening. Research could thus examine the conditions for the effective implementation of psychosocial interventions and disaster response services. In other words, future psychosocial disaster service research could seek to establish not just context-dependent but also more generalisable knowledge and theories about “what works, where and why across multiple contexts” (Damschroder et al., 2009).

Of course, central to these research questions is the issue of what outcomes these interventions and services are targeting, and how we should measure them. So while we have come a long way in our psychosocial responses to disaster, critical research questions still remain to be resolved in the future.

## Conclusion

Symposia such as those held by ISTSS/ESTSS in June 2013 that bring together both researchers and clinicians involved in disaster mental health responses provide a unique opportunity to consolidate learnings from disasters. Forums like these where disaster responses from different countries can be discussed create an environment where we can build upon past experience to ultimately improve future responses and minimise the mental health impact of disaster.
